# Meningothelial Hamartoma Masquerading as a Lipoma: A Case Report and Review of Literature

**DOI:** 10.7759/cureus.79732

**Published:** 2025-02-26

**Authors:** Saransh Gupta, Rekha Gupta

**Affiliations:** 1 Medicine, All India Institute of Medical Sciences, Bathinda, Bathinda, IND; 2 Pathology, Dr Lal PathLabs Limited, Jalandhar, IND

**Keywords:** ectopic, extracranial lipoma, meningothelial hamartoma, rare variant, scalp lump

## Abstract

Meningothelial hamartoma is a rare, benign scalp lesion that is often misdiagnosed as common soft tissue tumors such as lipomas. It is characterized by ectopic meningothelial elements and is classified as a type of cutaneous meningioma. Due to its rarity and histological overlap with other lesions, diagnosis requires histopathological examination and immunohistochemistry (IHC). A 36-year-old female presented with a painless, slow-growing lump on the left forehead persisting for 10 years. Magnetic resonance imaging (MRI) findings suggested a lipoma, and fine needle aspiration cytology (FNAC) indicated a fibro lipomatous lesion of neoplastic nature. It was surgically excised, and histopathology revealed mature adipose tissue with meningothelial cells. IHC showed epithelial membrane antigen (EMA) and vimentin positivity, confirming meningothelial hamartoma. The patient recovered well postoperatively, with no recurrence after one year. Meningothelial hamartoma is often misdiagnosed due to its resemblance to more common scalp tumors. Surgical excision is the mainstay of treatment, and IHC markers such as EMA and vimentin are essential for diagnosis. Given its potential hormonal influences and association with nevus sebaceous, further research is warranted. Clinicians should consider meningothelial hamartoma in atypical scalp lesions to ensure accurate diagnosis and appropriate management.

## Introduction

Scalp tumors are found globally, with the majority (93-99%) being benign, rather than malignant. Around 40-50% of benign scalp tumors consist of cysts, particularly trichilemmal cysts, while the remaining cases are mainly lipomas, accounting for approximately 30% [1]. Painless soft tissue masses on the scalp are frequently observed in clinical practice, with the most probable diagnoses being lipomas, epidermoid cysts, and sebaceous cysts. A scalp lipoma is a slow-growing, fatty tumor primarily composed of adipocytes. It typically presents as a smooth, round accumulation of subcutaneous fat with a rubbery texture upon palpation [2]. However, rare causes of soft tissue masses on the scalp, such as meningothelial hamartoma, can be easily overlooked and misdiagnosed as more common conditions such as lipomas. Meningothelial hamartoma, also known as rudimentary meningocele, is a scalp hamartoma characterized by ectopic meningothelial elements [3]. Other differentials for meningothelial hamartoma include dermoid cysts, which contain ectodermal and mesodermal elements; pilomatricoma, a benign calcifying tumor originating from hair follicle matrix cells; and neurofibromas, which are nerve sheath tumors that present as soft, well-circumscribed masses. Additionally, vascular anomalies such as hemangiomas or arteriovenous malformations may mimic soft tissue lesions on the scalp. Its diagnosis can only be confirmed through molecular techniques such as immunohistochemistry (IHC). Given its rarity and potential for misdiagnosis, we present a case that was initially mistaken for a lipoma but was ultimately identified as meningothelial hamartoma through histological examination, highlighting the importance of thorough pathological evaluation in these cases.

## Case presentation

**Figure 1:**
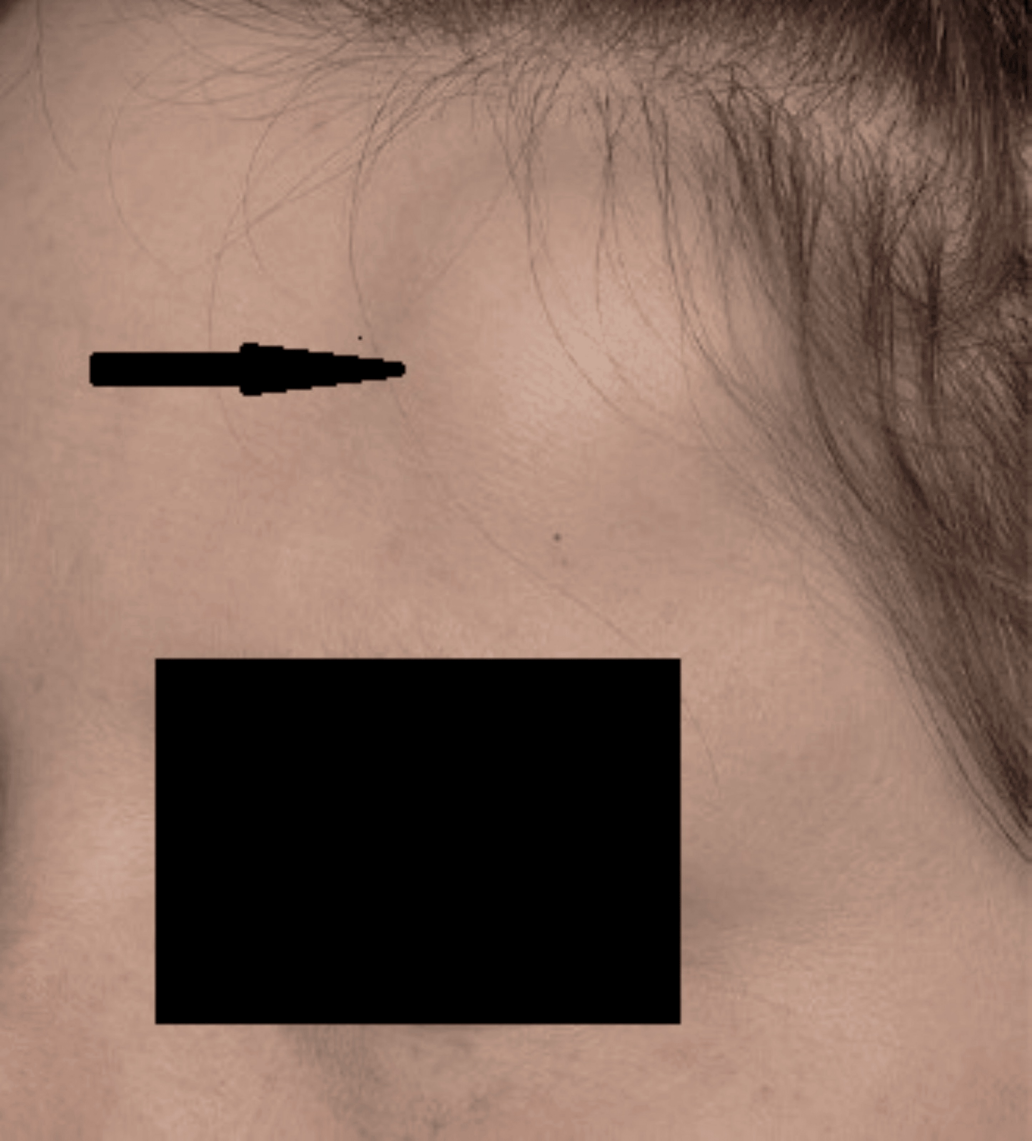
Gross image of the lump on the left forehead region of the patient (arrow)

A 36-year-old female presented in the outpatient department (OPD) with the chief complaint of a painless, enlarging lump on the left forehead region, from the past 10 years (Figure 1).

**Figure 2:**
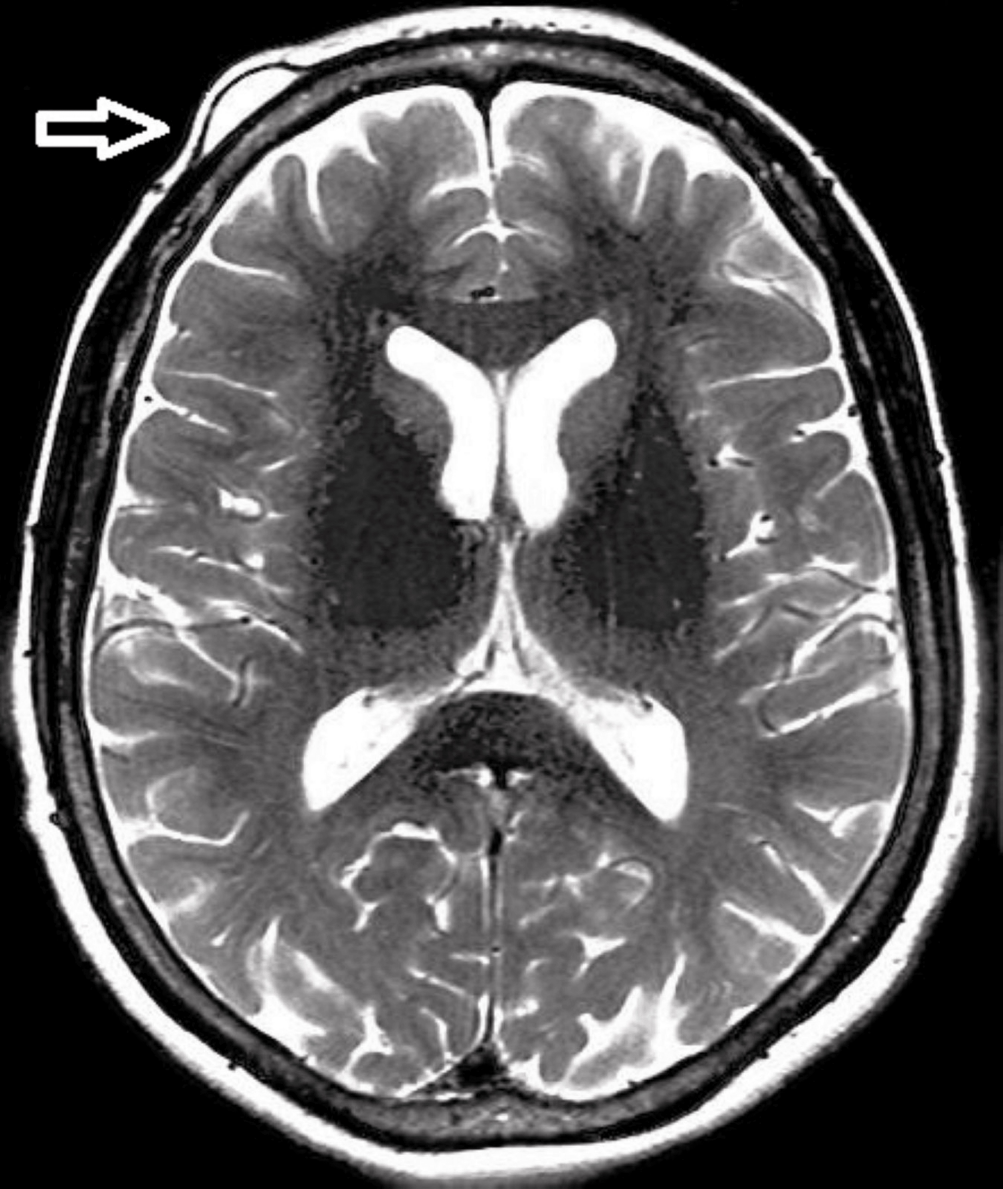
T2-weighted sequence of MRI of the head region showing oval-shaped hyperintensity in the left frontal region (arrow)

She had a negative history of fever and trauma, along with any negative family and substance abuse history. During physical examination, a lump measuring 3 x 2 cm was observed in the left frontal region. On palpation, it was soft in consistency, non-tender, and well-defined and had normal overlying skin. On blood investigations, the reports were normal, except for the reduced hemoglobin levels (8.6 mg/dL). On further investigation, the MRI scan of the brain revealed oval-shaped hyperintensity in the left frontal region, measuring 2.6 x 1.3 cm, with an intact cranium suggesting a lipoma. Upon contrast, the scan also revealed an area of T2 hyperintense and Flair hypointense bilateral frontal and basifrontal lobes marking encephalomalacia accompanied by mucosal thickenings in bilateral maxillary and ethmoid sinuses reflecting sinusitis (Figure 2).

**Figure 3:**
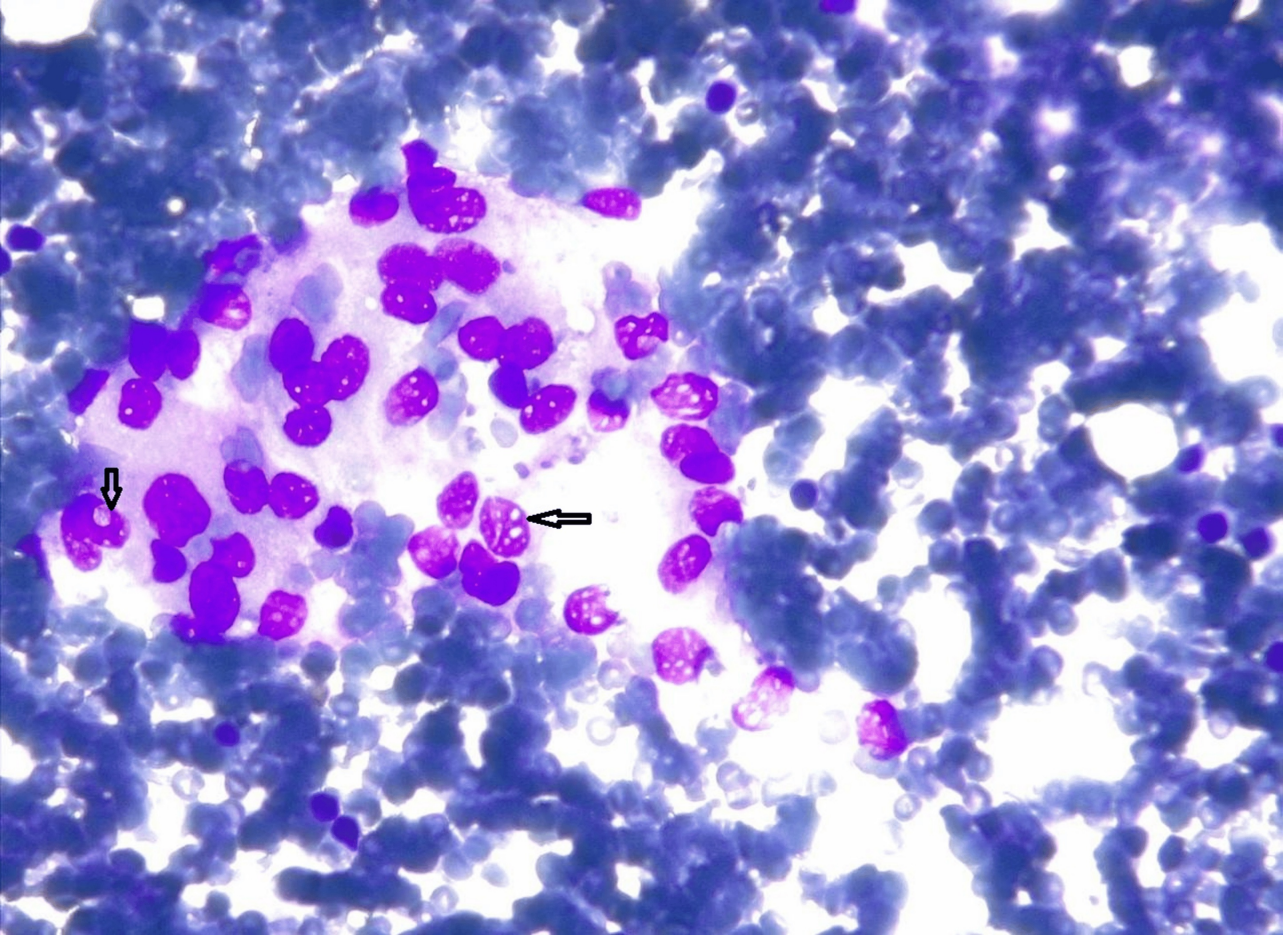
Cytology smear under 40x showing loose clusters of round-to-oval to epithelioid-like cells along with intranuclear inclusions (arrow)

For further diagnosis, fine needle aspiration cytology (FNAC) was performed. On aspiration, blood-mixed material was aspirated and stained with Giemsa, showing the presence of monomorphic sheets and clumps of round, oval-to-ovoid cells with moderate amphophilic cytoplasm. Nuclear grooving and intranuclear inclusion bodies were observed, with no signs of mitosis and only a few multinucleated giant cells, indicating a fibro lipomatous lesion of neoplastic nature (Figure 3).

**Figure 4:**
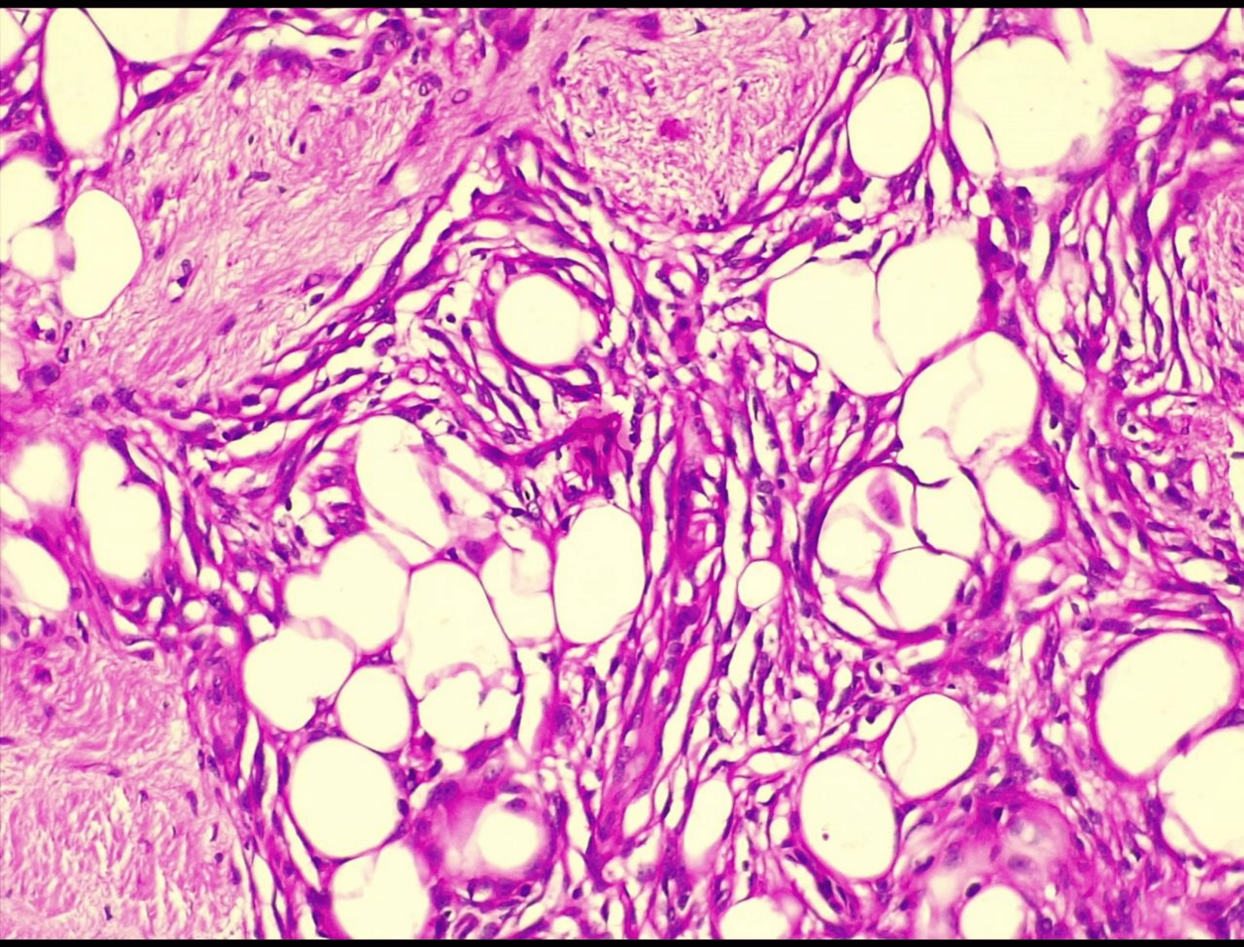
Hematoxylin- and eosin-stained histological section under 40x, showing whorls of meningothelial cells interspersed with adipocytes with adjacent fibrous tissue

Surgery was planned to remove the lump based on the clinical, radiological, and cytological impression of the lipoma. Total excision was performed. There was no cranial involvement on visual inspection. A gross examination revealed a pale, tan soft tissue mass covered with skin. The excised specimen was sent for histological confirmation. Histology of the specimen, stained with hematoxylin and eosin, revealed mature adipose tissue bands consisting of bundles and small nodules of dense collagen with the presence of meningothelial cells in nests and strands, giving a characteristic whorled appearance (Figure 4).

**Figure 5:**
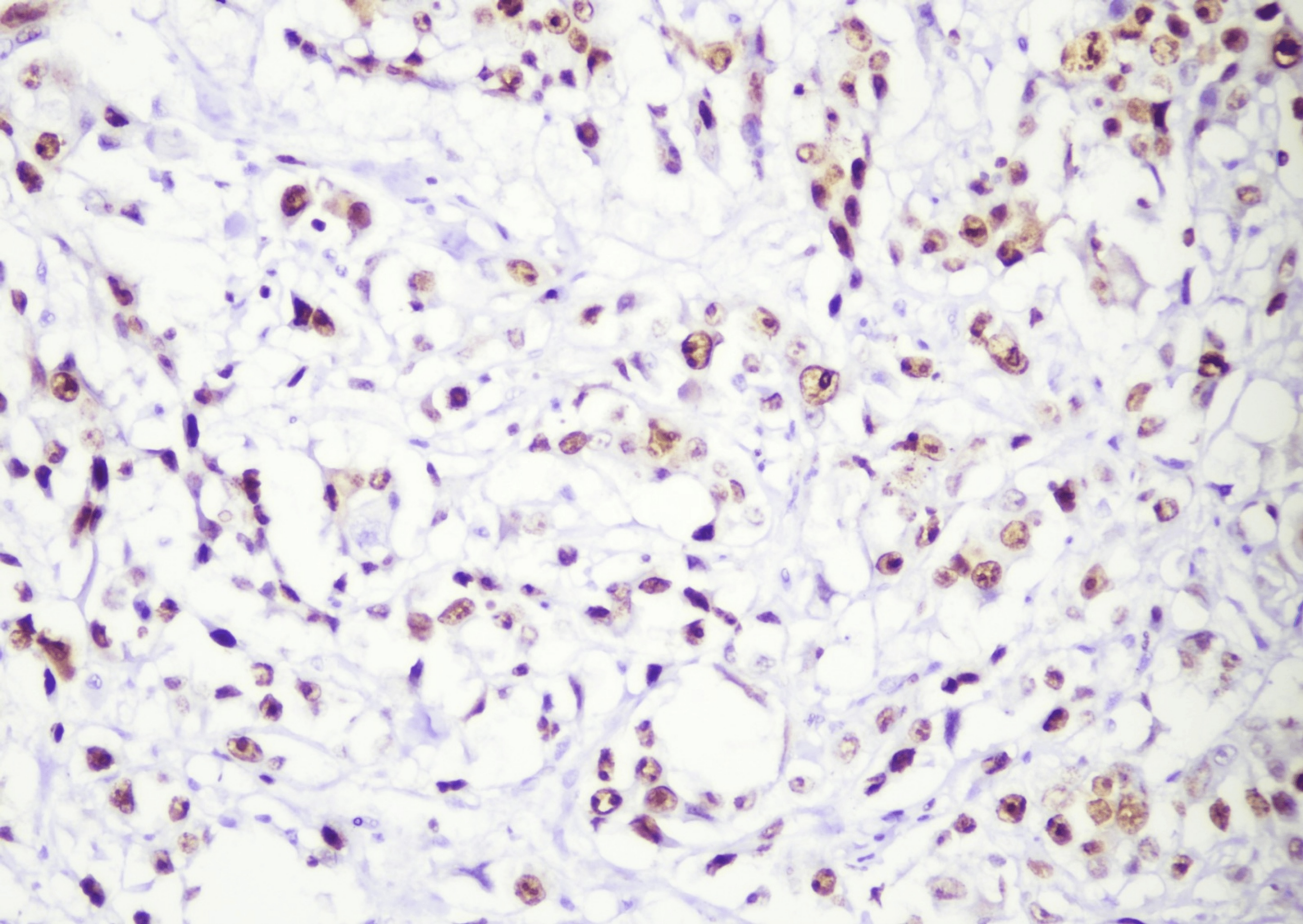
Immunohistochemical (IHC) staining showing positivity for vimentin

**Figure 6:**
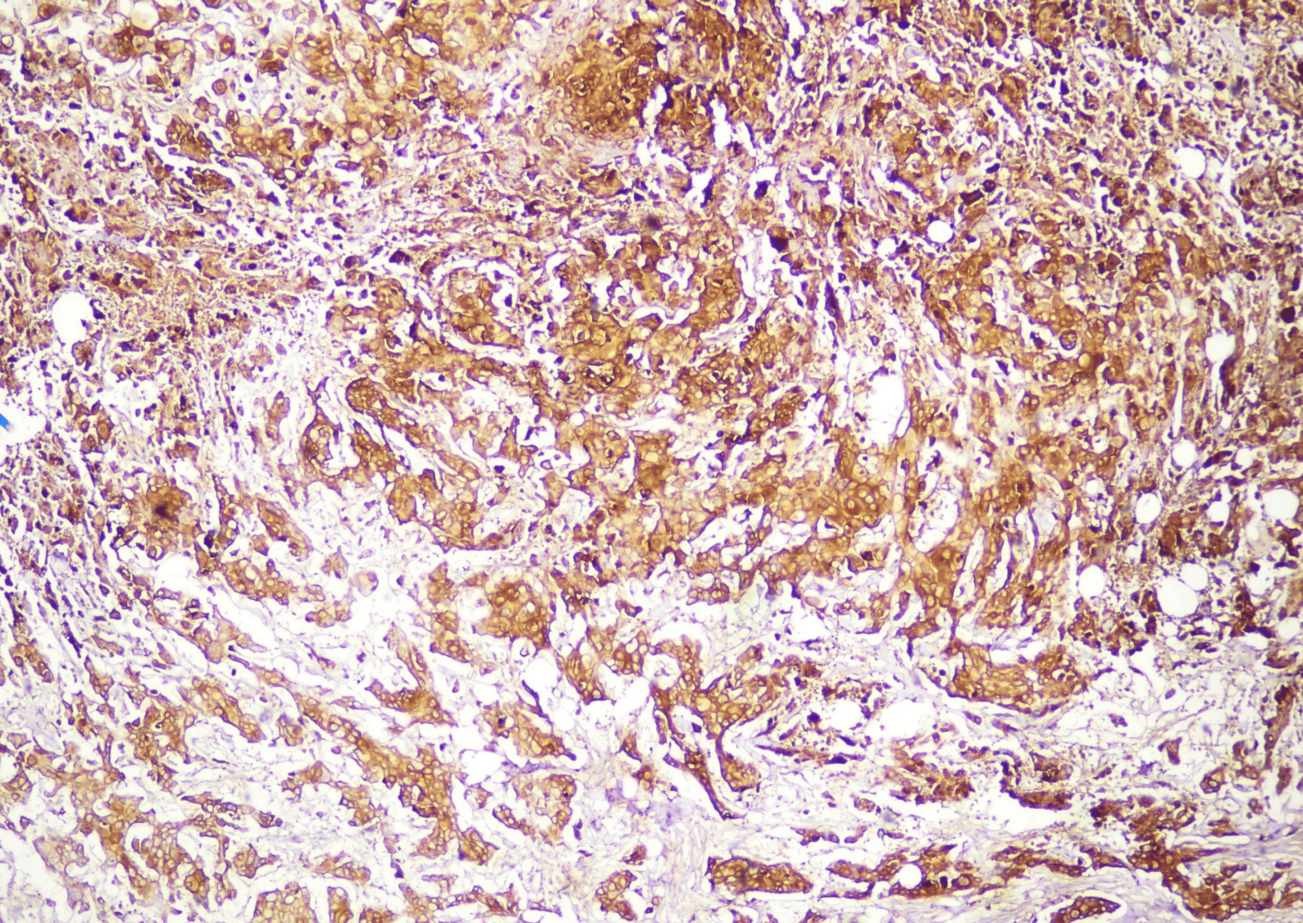
Immunohistochemical (IHC) staining showing strong positivity for epithelial membrane antigen (EMA)

To further confirm the diagnosis, IHC was performed. It was negative for human melanoma black-45 (HMB-45) and cytokeratin (CK), with very low immunoreactivity of Ki-67, ruling out malignant melanocytic lesions. It was also negative for glial fibrillary acidic protein (GFAP), ruling out the glial origin. IHC showed strong positivity for vimentin and epithelial membrane antigen (EMA) in the epithelioid cells (Figures 5-6). Progesterone receptors (PR) showed immunoreactivity in 20-25% of the lesional cells, confirming the diagnosis of meningothelial hamartoma. The patient was scheduled for a follow-up after two weeks. There were no signs of surgical site infection (SSI), and the lump has not recurred for a year. The patient resumed her daily lifestyle within four weeks.

## Discussion

Meningothelial hamartoma is a rare, benign lesion first described by Suster et al. in 1990 and initially classified as a type of cutaneous meningioma [4-6]. It was designated as type I (the congenital type), characterized by scattered meningothelial cells and psammoma bodies. The proposed pathogenesis involves ectopic displacement of meningothelial cell precursors during embryogenesis or, alternatively, as a consequence of an obliterated meningocele. Several cases have since been reported, further expanding our understanding of this condition. Suster et al. described a 19-year-old male in whom molecular genetic testing and IHC revealed no association with neoplasia [4]. Similarly, Curran-Melendez et al. reported a case of a nine-year-old child with a lesion in the posterior region, which is a more commonly affected site. Their study reinforced that meningothelial hamartoma predominantly affects the young population. They also noted that lesions exhibit a variety of colors, including tan-grey, red, flesh-colored, and amelanotic, and categorized them into fixed and mobile masses, both of which have a very low recurrence rate [7].

Histopathologically, David et al. described these lesions as containing proliferative connective tissue, epithelioid cells forming interanastomosing vascular channels, and meningothelial elements. Given the potential for a persistent connection with the central nervous system (CNS), they emphasized the necessity of comprehensive preoperative evaluation [8]. This remains a critical consideration in clinical practice to avoid complications during surgical excision. Differential diagnoses for meningothelial hamartoma are broad and include angiosarcoma, spindle cell haemangioendothelioma, epithelioid haemangioma, giant cell fibro blastoma, intravascular papillary endothelial hyperplasia, and spindle cell lipoma [8]. Given this wide range of possibilities, histological and immunohistochemical analysis remains essential for accurate diagnosis. Management of meningothelial hamartoma primarily involves surgical excision, which remains the cornerstone of treatment. A review of reported cases suggests that recurrence is extremely rare, with only one documented case [9]. Additionally, Biagiotti et al. reported a case of meningothelial hamartoma associated with nevus sebaceous, necessitating early surgical intervention. This association suggests that in cases where concomitant skin lesions are present, a multidisciplinary approach may be required to optimize treatment strategies [6]. Meningothelial hamartoma can be mistaken for various benign scalp lesions, including dermoid cysts, epidermoid cysts, pilomatricomas, neurofibromas, and vascular anomalies such as hemangiomas. Dermoid and epidermoid cysts contain keratinized epithelium and lack meningothelial cells, unlike meningothelial hamartoma, which shows meningothelial whorls and psammoma bodies. Pilomatricomas exhibit basaloid cells with ghost cells, while neurofibromas display spindle cells positive for S-100. Hemangiomas show proliferating blood vessels lined by CD31/CD34-positive endothelial cells. In contrast, meningothelial hamartomas stain positively for EMA, vimentin, and PR, confirming their meningothelial origin. Grossly, they appear as well-circumscribed, firm masses without cystic or vascular structures. Given the rarity of this condition, each new case report provides valuable insights into its clinical presentation, histopathology, and management. Our case contributes to the limited literature on meningothelial hamartoma and highlights the importance of considering it as a differential diagnosis for cutaneous lesions, particularly in young patients.

## Conclusions

Meningothelial hamartoma is a rare, often misdiagnosed scalp lesion that mimics common conditions such as lipomas. This case highlights the importance of histopathological and immunohistochemical analysis for accurate diagnosis. While surgical excision remains the definitive treatment, preoperative imaging is crucial to rule out intracranial involvement. Given its rarity, further research is needed to explore potential genetic and hormonal influences, as PR positivity suggests a possible role of hormonal regulation. Additionally, its association with nevus sebaceous warrants investigation into developmental anomalies. Clinicians should maintain a high index of suspicion in atypical cases and utilize IHC markers such as EMA and vimentin for confirmation. Although recurrence is rare, periodic follow-up may be beneficial in select cases. This report contributes to the limited literature, emphasizing early recognition, accurate diagnosis, and optimal management of this uncommon entity.
